# Physical inactivity in times of a pandemic: another curve to flatten

**DOI:** 10.1007/s12471-021-01576-5

**Published:** 2021-04-14

**Authors:** H. T. Jorstad, J. J. Piek

**Affiliations:** Department of Cardiology, Heart Centre, Amsterdam UMC, location AMC, University of Amsterdam, Amsterdam, The Netherlands

Worldwide, ‘lockdown’ measures to limit the spread of severe acute respiratory coronavirus‑2 (SARS-CoV-2) disease (COVID-19) have been shown to negatively influence levels of physical activity, exercise and sedentary behaviour [1]. Patients with cardiovascular disease (CVD) are of particular concern, as the severity and risk of developing complications from COVID-19 have been shown to be markedly higher in individuals with underlying CVD or CVD risk factors [2]. Remaining physically healthy and active could potentially reduce some of this risk [3]. Conversely, lockdown measures may form a barrier to continued physical exercise and stimulate inactivity, with the associated additional negative effects on risk of events for the underlying CVD [4].

In this issue of *the Netherlands Heart Journal,* van Bakel and colleagues [5] demonstrate a complex relationship between changes in self-reported physical activity and sedentary behaviour in 1565 Dutch CVD patients during the first lockdown in the Netherlands in April 2020. In short, when compared to 2018, they show that levels of moderate-to-vigorous physical activity actually increase (from 1.6 to 2.0 h/day), yet time spent exercising decreases (from 1.0 to 0.0 h/week) and sedentary time increases (from 7.8 to 8.9 h/week), resulting in a net reduction of habitual physical activity. Based on these findings, the authors argue that ‘interventions are needed to increase physical activity levels and reduce sedentary time’, and suggest telerehabilitation as a possible solution.

These findings highlight a number of important challenges, but also a number of opportunities. First, the majority of individuals with CVD make an effort to remain physically active and, despite lockdown measures, actually succeed to some degree, through activities such as walking and performing odd jobs. However, when deprived of the setting where they habitually exercise, time spent exercising decreases. Second, when individuals with CVD are confined to their homes, sedentary time increases. This should not be surprising, as most homes are designed to stimulate time spent sitting and conventionally include multiple labour-saving devices [6]. Even the most physically active among us—healthy children—have been shown to spend 67% of their time at home sitting [7]. Finally, the current pandemic and the measures taken are unprecedented, and the long-term consequences of lockdown measures on physical activity levels are unknown. When lockdowns are (hopefully) permanently ended, it is unclear whether this will lead to a resurgence of sports and exercise, or whether the decrease in time spent exercising will lead to long-term unfavourable lifestyle changes.

With a trend towards yearly increases in total time spent sitting, with more pronounced increases in older individuals [8], improving physical activity levels and reducing sedentary time remain two of the most central challenges of the modern world, regardless of pandemics or CVD status. In a society where the living environment promotes sedentary behaviour [9], health care professionals alone, whether through conventional cardiac rehabilitation or telerehabilitation, will not be able to flatten the curve of increasing inactivity. Concerted action, including contributors from outside health care, will be required. The home-based office could be equipped with a standing desk; long digital meetings could be interrupted by a plenary break with physical activity. Time spent exercising should not be synonymous with going to a gym or a physiotherapist, or with cost; running shoes and a bicycle should suffice and perhaps be a part of telerehabilitation or an insurer’s programme. While easy to say in hindsight, the first Dutch lockdown might have been used as an opportunity to emphasise that remaining physically active and healthy, and curbing risk factors for CVD, is a central part of the preventive efforts not only for CVD, but also for reducing the risk of a severe course of COVID-19.

When the nation is unlocked, priority should perhaps be given to achieving levels of sports and exercise *higher* than those pre-lockdown, not only by health care professionals, but also by scientific policy makers, professional societies and funding bodies. Lack of exercise and physical inactivity remain central challenges of our society, resulting in a curve of inactivity that we collectively and chronically need to flatten. In the end, whether you engage in physical activity yourself, work to stimulate individuals with CVD to be physically active, or both: *good things come to those who sweat*.
